# Impact of the medial displacement calcaneal osteotomy on foot biomechanics: a systematic literature review

**DOI:** 10.1007/s00402-024-05267-9

**Published:** 2024-03-30

**Authors:** Gunnar Mueller, Karl-Heinz Frosch, Alexej Barg, Carsten Schlickewei, Hanneke Weel, Nicola Krähenbühl, Matthias Priemel, Elena Mueller

**Affiliations:** 1 Department of Trauma and Orthopaedic Surgery, Hospital Itzehoe, Itzehoe, Germany; 2 Department of Trauma and Orthopaedic Surgery, University Medical Center Hamburg-Eppendorf, Martinistrasse 52, 20246 Hamburg, Germany; 3 Department of Trauma Surgery, Orthopaedics, and Sports Traumatology, BG Hospital Hamburg, Hamburg, Germany; 4 Bergman Clinics, Department of Orthopedics Arnhem, Arnhem, CV The Netherlands; 5 Department of Orthopaedics, University Hospital Basel, Basel, Switzerland

**Keywords:** Progressive collapsing foot deformity, Flatfoot deformity, Medial displacement calcaneal osteotomy, Biomechanics, Systematic literature review

## Abstract

**Introduction:**

Progressive collapsing foot deformity (PCFD), formally known as “adult-acquired flatfoot deformity” (AAFFD), is a complex foot deformity consisting of multiple components. If surgery is required, joint-preserving procedures, such as a medial displacement calcaneal osteotomy (MDCO), are frequently performed. The aim of this systematic review is to provide a summary of the evidence on the impact of MDCO on foot biomechanics.

**Materials and methods:**

A systematic literature search across two major sources (PubMed and Scopus) without time limitation was performed according to the Preferred Reporting Items for Systematic Review and Meta-Analyses (PRISMA) criteria. Only original research studies reporting on biomechanical changes following a MDCO were included. Exclusion criteria consisted of review articles, case studies, and studies not written in English. 27 studies were included and the methodologic quality graded according to the QUACS scale and the modified Coleman score.

**Results:**

The 27 included studies consisted of 18 cadaveric, 7 studies based on biomechanical models, and 2 clinical studies. The impact of MDCO on the following five major parameters were assessed: plantar fascia (*n* = 6), medial longitudinal arch (*n* = 9), hind- and midfoot joint pressures (*n* = 10), Achilles tendon (*n* = 5), and gait pattern parameters (*n* = 3). The quality of the studies was moderate to good with a pooled mean QUACS score of 65% (range 46–92%) for in-vitro and a pooled mean Coleman score of 58 (range 56–65) points for clinical studies.

**Conclusion:**

A thorough knowledge of how MDCO impacts foot function is key in properly understanding the postoperative effects of this commonly performed procedure. According to the evidence, MDCO impacts the function of the plantar fascia and Achilles tendon, the integrity of the medial longitudinal arch, hind- and midfoot joint pressures, and consequently specific gait pattern parameters.

**Supplementary Information:**

The online version contains supplementary material available at 10.1007/s00402-024-05267-9.

## Introduction

Progressive collapsing foot deformity (PCFD), also known as “adult-acquired flatfoot deformity” (AAFFD), is a complex foot deformity consisting of multiple components: (1) peritalar subluxation resulting in foot deviation in various planes (e.g., hindfoot valgus, talar plantarflexion, forefoot supination); (2) abduction deformity at the level of the midfoot; and (3) forefoot varus with the first ray elevated above the fifth metatarsal [1, 2]. The etiology is still unclear, but generally involves soft tissue degeneration associated with dysfunction of the tibialis posterior tendon (PTT) [3, 4].

If surgery is required, joint-preserving procedures, such as a medial displacement calcaneal osteotomy (MDCO), are frequently performed. Calcaneal osteotomies as part of a bony reconstruction address hindfoot valgus and may also impact midfoot abduction [5–7]. The MDCO, first described by Gleich in 1893 [8], involves a medial translation of the entire tuber calcanei. As the lever arm of the Achilles tendon changes, such an osteotomy may have a significant effect on foot function.

Several studies assessed the impact of MDCO on foot biomechanics. Nevertheless, the understanding of MDCO on foot function is currently limited. Therefore, the aims of this systematic review are: (1) to perform a systematic literature search on studies assessing the impact of MDCO on foot biomechanics; (2) to provide an overview of the current knowledge on the effect of MDCO on foot biomechanics; and (3) to grade these studies according to their methodological quality.

## Materials and methods

### Search strategy and study selection

The original protocol for this systematic literature review was registered on PROSPERO, the international prospective register of systematic reviews (CRD42022270180), after performing a search on the CRD database to find out whether this review was already registered. The electronic MEDLINE database via PubMed and Scopus were systematically searched. The search was performed on the 5th of July 2021, with a final update on the 25th of January 2023. The following search algorithm was used: (calcan* [ALL] AND osteotomy [ALL]) OR (sliding AND calcan*) OR (calcaneus [MeSh] AND osteotomy [MeSh]). There were no limitations on the type of journal or article publication date. Only publications in English were included. The article bibliographies were also reviewed. Bidirectional citation search was used including backward and forward citation search methods [9]. The systematic literature search was conducted independently by three authors (GM, CS and EM) according to the PRISMA (Preferred Reporting Items for Systematic Review and Meta-Analysis) guidelines [10]. Studies were included if they were original research reporting data on biomechanical changes following a MDCO. Exclusion criteria included review articles, case studies, and studies not written in English. The study selection process was performed independently by three observers (EM, GM, CS). In case of a disagreement, a final decision was made by group consensus.

### Data extraction and quality assessment

The following data from the included studies were extracted: type of study, number of cadaveric specimens / number of biomechanical models / number of feet, age, biomechanical testing method (if appropriate), and the impact on foot biomechanics as primary outcome parameters. In addition, proposed therapeutic and/or surgical consequences were collected as secondary outcome parameters. Data extraction was independently performed by two review authors (GM and EM). The methodological quality of the studies including cadavers was assessed using the QUACS scale (Quality Appraisal for Cadaveric Studies; Table 1) [11]. Two reviewers (GM and CS) independently examined each included study using the checklist consisting of 13 items and reported scores as percentages. These scores were pooled and reported as an average. Clinical studies were graded using the modified Coleman score *(*Table 2*)* [12]. The modified Coleman score was applied by two independent reviewers (GM and CS). Each study was assessed for study size, follow-up time, percentage of patients with follow-up, number of interventions, study type, diagnostic certainty, description of surgical method/rehabilitation, outcome criteria/assessment process, and patient selection process.

**Table 1:** Items and scoring criteria of the QUACS scale

Item	When to score ‘yes’
Objective stated	The study’s aims are clearly stated and hypotheses presented
Information about sample is included	Age (range/mean and standard deviation), gender and sample size are stated
Applied methods are described comprehensibly	Clearly structured, detailed outline of the study protocol and the process of dissection
Study reports condition of the examined specimens	Specimens’ status (healthy vs. injured, etc.) is stated (embalmed cadavers = type of solution is reported)
Education of dissecting researchers is stated	Study reports knowledge/professional state and/or experience of the investigator
Findings are observed by more than one researcher	Stated clearly, that two or more persons independently made the observations
Results presented thoroughly and precise	Results described with clear structure, and including, figures, illustrations or tables
Statistical methods appropriate	Correct choice and application of statistical data analysis
Details about consistency of findings are given	Information on number or percentage of cases the observation was made in
Photographs of the observations are included	Photographs of the key observations (e.g., continuities) with precise labels are included
Study is discussed within the context of current evidence	Other relevant trials relating to the field of study are stated and discussed
Clinical implications of the results are discussed	Similar studies are reported, added knowledge and its relevance to the field are pointed out
Limitations of the study are addressed	Weaknesses and methodological shortcomings are reported

**Table 2:** Modified coleman methodology score

Part A - only one score to be given for each of the seven sections		
1. Study size - number of patients	> 51	10
	31–50	7
	11–30	4
	< 10 or not stated	0
2. Mean follow-up	> 6 years	5
	2.1-6 years	3
	< 2 years, not stated or unclear	0
3. Percent of patients with follow-up	> 90%	5
	80–90%	3
	< 80%	0
4. Number of interventions per group	One intervention in all patients	10
	Multiple interventions but consistent	5
	Inconsistent, unclear, not reported	0
5. Type of study	Randomized control trial	15
	Prospective cohort study	10
	Retrospective cohort study	0
6. Diagnostic certainty	In all	5
	In > 80%	3
	In < 80%, instated, or unclear	0
7. Description of surgical technique	Technique stated with details	5
	Technique named without elaboration	3
	Not stated or unclear	0
8. Description of postoperative rehabilitation	Described, > 80% compliance	5
	Described, 60–80% compliance	3
	Not reported, < 60% compliance	0
Part B - scores may be given for each option in each of the three sections		
1. Outcome criteria	Outcome measures clearly defined	2
	Timing of outcome assessment clear	2
	Outcome criteria with good reliability	3
	Outcome with good sensitivity	3
2. Procedure for assessing outcomes	Subjects recruited	5
	Independent investigator	4
	Written assessment	3
	Patient centered data collected	3
3. Description of subject selection process	Selection criteria reported and unbiased	5
	Recruitment rate reported and > 80%	5
	Eligible subjects not included accounted for	5
	Total	100

### Statistical analysis

The data were processed descriptively; therefore, no meta-analysis was performed. Patient demographic characteristics (number of patients/ feet, patient age and sex) were summarized. Weighted median scores were calculated for the modified Coleman and QUACS scores. Data analysis was performed using IBM SPSS Statistics Version 26.0 (IBM Corp., Armonk, NY, USA).

## Results

**Fig. 1:**
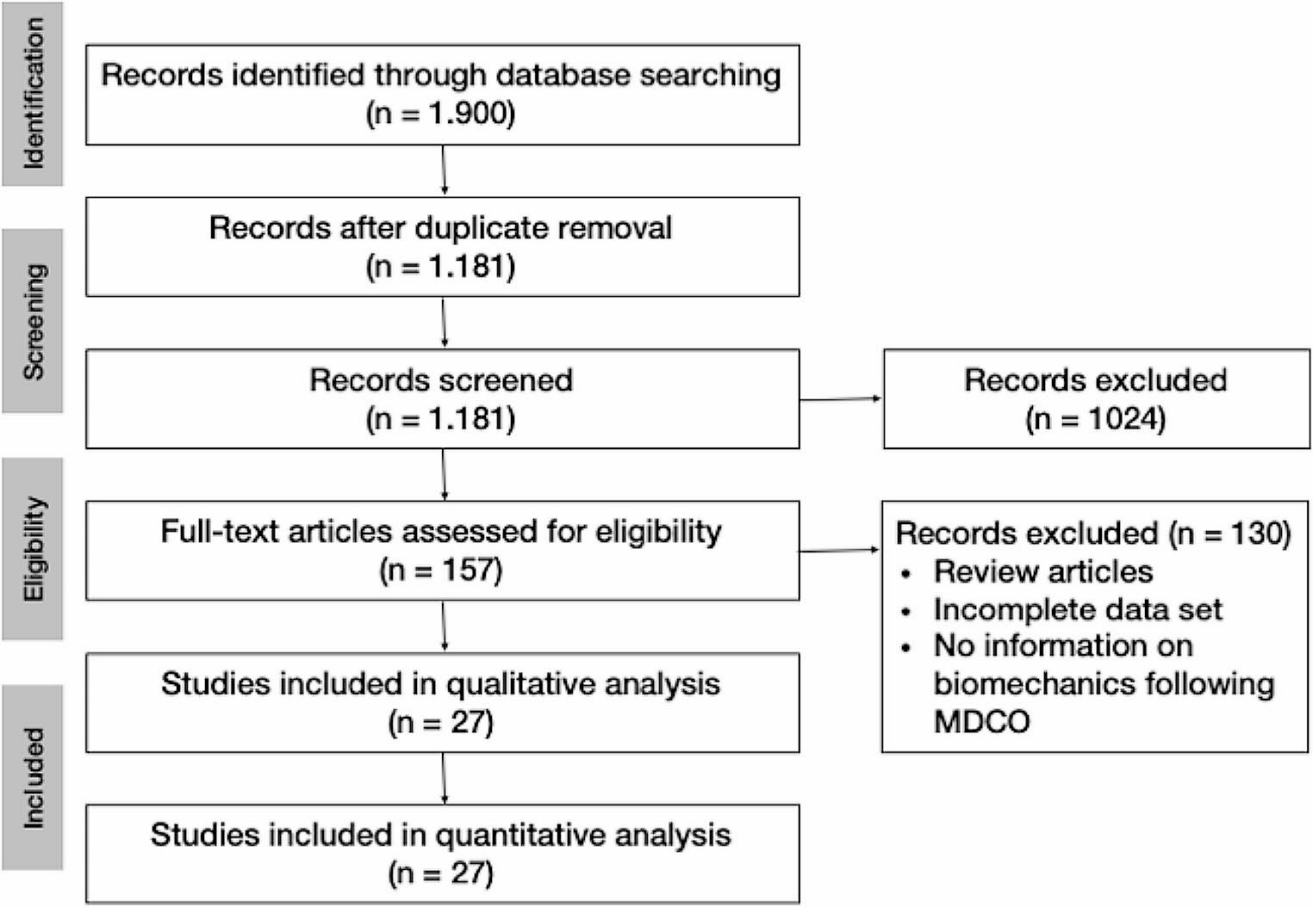
Flowchart depicting the strategy used to select relevant studies

The initial database screening resulted in 1,900 studies. After removing all duplicates and screening of titles and abstracts, 157 studies were eligible for full-text review. After exclusion of 130 studies according to our exclusion criteria (review articles, incomplete data set, no information on foot biomechanics), 27 studies were ultimately included in the final analysis. The selection process was performed according to “Preferred Reporting Items for Systematic Review and Meta-Analyses” (PRISMA) and is shown in Fig. 1 [10].

### Study characteristics

The 27 included studies were published between 1995 and 2020 and contained 18 cadaveric studies, 7 studies using biomechanical models, and 2 clinical studies (Table 3). The 18 cadaveric studies included 185 cadavers (188 feet) with a pooled mean age of 65 years (range 43–96 years; age defined in 6 studies), comprised of 13 (50%) female and 13 (50%) male patients (gender defined in 4 studies). Within the 7 studies using biomechanical models, the models were based either on cadavers or MRI / CT scan datasets (including 2 Finite-Element-models). The 2 clinical studies evaluated a total of 93 patients (96 feet) with a pooled mean age of 58 years (range 43–79), comprised of 12 (13%) male and 81 (87%) female patients. Studies including cadavers had a pooled mean QUACS score of 65% (range 46–92%). The pooled mean Coleman score of the clinical studies was 58 (range 56–65) points (Table 3*)*. Within the included studies, 4 were financially supported by different grants.

**Table 3:** Study type characteristics, modified Coleman methodology score and QUACS Score of 27 studies addressing biomechanics of medial displacement calcaneal osteotomy

Study	Type	QUACS Scale	Coleman Score
Fairbank et al. (1995)	Cadaver	54%	
Resnick (1995)	Cadaver	54%	
Steffensmeier et al. (1996)	Cadaver	62%	
Horton et al. (1998)	Cadaver	54%	
Michelson et al. (1998)	Cadaver	54%	
Thodarson et al. (1998)	Cadaver	62%	
Deland et al. (1999)	Cadaver	62%	
Otis et al. (1999)	Cadaver	62%	
Arangio et al. (2001)	Biomechanical Model	46%	
Davitt et al. (2001)	Cadaver	62%	
Nyska et al. (2001)	Cadaver	62%	
Sung et al. (2002)	Cadaver	54%	
Hadfield et al. (2003)	Cadaver	62%	
Hadfield et al. (2005)	Cadaver	55%	
Havenhill et al. (2005)	Cadaver	69%	
Scott et al. (2007)	Cadaver	69%	
Arangio et al. (2009)	Biomechanical Model	62%	
Marks et al. (2009)	Clinical Study		65
Iaquinto et al. (2011)	Biomechanical Model	62%	
Reilingh et al. (2011)	Biomechanical	77%	
Schuh et al. (2013)	Clinical Study		56
Zanolli et al. (2014)	Cadaver	77%	
Spratley et al. (2015)	Biomechanical Model	77%	
Patrick et al. (2016)	Cadaver	77%	
Smith et al. (2017)	Biomechanical Model	62%	
Wang et al. (2018)	Biomechanical Model	92%	
Malik et al. (2020)	Cadaver	77%	

### Impact on the plantar fascia

Within the included studies, 6 assessed the impact of MDCO on the plantar fascia *(*Table 4*)*. Thordarson et al. showed in a cadaveric study that a transection of the plantar fascia does not affect the corrective potential of MDCO [13]. Iaquinto et al. demonstrated an 80% increase of plantar fascia strain in AAFFD patients, with a significant decrease following MDCO [14]. Similar findings were evident in the studies published by Smith et al. and Spratley et al. [15–17]. Horton and colleagues demonstrated that a 1 cm MDCO resulted in an average of 1.2 ± 0.5 mm shortening of the plantar fascia. This finding was confirmed by Sung et al. [18].

**Table 4:** Studies reporting of impact of a MDCO on the plantar fascia

Author	Type	Feet	Age (Y)	Methods	Results	Consequences
Horton (1998) [17]	Cadaver	*N* = 9	NR	Flatfoot model. Axial load to 400 N, measurement of plantar fascia strain. After MDCO and after lateral column lengthening measurement of fractional length changes in plantar fascia through calcaneocuboid joint.	No tightening of plantar fascia with MDCO / lateral column lengthening. Plantar fascia significantly less taut with MDCO / lateral column lengthening. Lateral column lengthening produced significantly looser plantar fascia than MDCO.	NR
Iaquinto (2011) [15]	Biomechanical Model	*N* = 1	NR	Cadaveric anatomy captured by CT imaging, imported to modeling software. Ligament stiffnesses modified to reflect stage II flatfoot deformity, followed by corrective osteotomies.	80% increase of plantar fascia strain under flatfoot condition. 1 cm MDCO: significant drops plantar fascia strain (87% of normal value).	This study suggests that MDCO and lateral column procedures can lead to overcorrection of the deformity.
Smith (2017) [16]	Biomechanical Model	*N* = 6	NR	Patient specific computational models (created from MRI) followed by procedures incorporated a TT and hindfoot procedures (MDCO and lateral column procedures: Evans osteotomy, calcaneocuboid distraction arthrodesis, Z osteotomy, or combination).	With exception to lateral bands of the plantar fascia and middle spring ligament, the strain present in the plantar fascia, spring ligament, and deltoid ligaments decreased after all procedures.	The combination MDCO and Evans as well as MDCO and Z osteotomy were shown to provide the greatest amount of correction for forefoot abduction and hindfoot valgus.
Spratley (2015) [18]	Biomechanical Model	*N* = 6	50 (26–69)	Flatfoot models via MRI. Surgical effect of TT with / without MDCO quantified using X-rays and pedo-barography.	12 of 14 measures increased following TT only. Largest changes for TT combined with a 10 mm MDCO.	NR
Sung (2002) [19]	Cadaver	*N* = 13	NR	Cadaver specimens loaded in custom testing apparatus. Measurement of PTT force required to achieve early heel rise in intact foot compared to requirements following MDCO and posterior distraction osteotomy.	Force required of PTT to achieve early heel rise decreased from 399 ± 50 N in intact foot to 328 ± 78 N after MDCO and from 206 ± 122 N after posterior distraction.	NR
Thordarson (1998) [14]	Cadaver	*N* = 7	NR	Record of angular relationships between 1st metatarsal and talus with motion analysis system in transverse, sagittal, coronal planes with / without flatfoot condition / MDCO and division of plantar fascia.	Significant deformity increases after division of plantar fascia. Correction of flatfoot deformity in all planes with MDCO with / without intact plantar fascia.	The division of the plantar fascia does not have any effect on the corrective potential of MDCO.

N = number; Y = years; NR = not reported; TT = tendon transfer; PTT = posterior tibial tendon

### Impact on the medial longitudinal arch

The impact of MDCO on the medial longitudinal arch was evaluated in 9 studies *(*Table 5*)*. Otis et al. analyzed the length of the spring ligament under a load of 100 N in a cadaver study. The length was comparable before and after MDCO [19]. Sung and colleagues confirmed these results in their cadaveric study [18]. Arangio et al. used a 3D biomechanical multisegmental model to analyze the effect of MDCO on the excess forces in the medial longitudinal arch. They demonstrated that MDCO with 10 mm of displacement results in a substantial shift of excess forces toward the lateral side and hereby decreases the load on the medial arch [20]. Hadfield and colleagues confirmed this finding in a cadaver study [21]. In a further study they also showed a 0.5 cm superior translation of the osteotomy to result in a greater load reduction of the first and second metatarsals [22]. Spratley et al. also showed a change in plantar force distribution with a reduce in load of the medial forefoot [17]. Smith et al. proved with patient specific computational models a decrease of plantar fascia, spring ligament, and deltoid ligament sprain loads [15]. Zanolli et al. demonstrated in a cadaveric study that an additional reconstruction of the spring ligament does not change the effect of MDCO [23]. Resnick et al. analyzed the deltoid ligament force under axial loading. Different clinical and surgical conditions were simulated. A decreased force could be detected when MDCO was combined with a triple arthrodesis [24].

**Table 5:** Studies reporting of impact of a MDCO on the medial longitudinal arch

Authors	Type	Feet	Age (Y)	Methods	Results	Consequences
Arangio (2001) [21]	Biomechanical Model	*N* = 1	NR	Biomechanical and multisegmental model in conjunction with experimental literature data. Analysis of response of normal foot, flatfoot and flatfoot with MDCO to load and calculation of distribution of support among metatarsal heads and moment of various joints.	Flatfoot: shift of distribution of support from lateral to medial- decreasing support 5th metatarsal (11–1%), increasing support 1st metatarsal (12–22%) and increasing moment talo-navicular joint (20 to 28 Nm). 10 mm MDCO: shift back to lateral side (11% 5th metatarsal, 13% 1st metatarsal, decreasing moment talo-navicular joint to 18 Nm). 10 mm MDCO in a PCFD model decreases load on medial arch.	NR
Arangio (2009) [22]	Biomechanical Model	*N* = 1	77	Analysis healthy foot, flatfoot, 10 mm MDCO, and FDLT. CAT scan and direct linear transformation was performed.	Flatfoot: portion of body weight supported by rays of medial arch increased, portion of body weight supported by 5th metatarsal decreased. MDCO reduced load on 1st and 2nd metatarsal, increased load on 5th metatarsal; reduced load on ligaments supporting talo-navicular joint and joints of medial arc.	NR
Hadfield (2003) [23]	Cadaver	*N* = 14	NR	Axial loading in neutral and dorsiflexion for 1 cm MDCO.	Average pressure over 1st and 2nd metatarsal decreased after MDCO. Significant increase in average pressure over medial and lateral aspect of heel and substantial inversion of forefoot.	MDCO increases forefoot varus but unloads 1st and 2nd metatarsal.
Hadfield (2005) [24]	Cadaver	*N* = 28	64 (48–82)	Axial load on a load frame device to assess effects of 1 cm MDCO in conjunction with 0.5 cm / 1 cm superior translation on foot pressures.	0.5 cm superior translation: greater offloading of 1st and 2nd metatarsals without increasing lateral forefoot or heel pressures. 1 cm superior translation continued to unload 1st/2nd metatarsal but lateral forefoot / midfoot pressures increased.	Recommendation of incorporation of a 5 mm superior translation when performing MDCO.
Otis (1999) [20]	Cadaver	*N* = 9	NR	Cadaver feet subjected to vertical loads. Monitoring of spring ligament length and change in length per unit of applied load.	Length of the spring ligament comparable before versus after MDCO.	NR
Resnick (1995) [26]	Cadaver	*N* = 6	NR	Axial load and test of deltoid ligament force. Simulation of 5 conditions. 1: Intact, tensioned PTT; 2: Ruptured PTT; 3: Triple arthrodesis with ruptured PTT; 4: Lateral displacement calcaneal osteotomy in combination with triple arthrodesis and ruptured PTT; 5: MDCO in combination with triple arthrodesis and ruptured PTT.	Test 1: control value of deltoid ligament force; Test 2: averaged 97% more deltoid ligament force than Test 1; Test 3: similar to Test 1 but 47% less than Test 2; Test 4: 76% more than with intact PTT and 69% more than with triple arthrodesis alone; Test 5: force seen in deltoid ligament averaged 23% less than that seen with intact PTT and 61% less than that seen with ruptured PTT and 56% less than that seen in the lateral displacement calcaneal osteotomy.	Patients with PTT rupture and peritalar subluxation have suboptimal results after triple arthrodesis (elevated forces in the deltoid ligament resulting in laxity). MDCO in combination with triple arthrodesis may be viable treatment when hindfoot cannot be positioned properly.
Smith (2017) [16]	Biomechanical Model	*N* = 6	NR	See Table 4.	With exception of lateral bands of the plantar fascia and middle spring ligament, the strain present in the plantar fascia, spring, and deltoid ligaments decreased.	See Table 4.
Spratley (2015) [18]	Biomechanical Model	*N* = 6	50 (26–69)	See Table 4.	Largest improvements for TT and MDCO. Alterations in spring, deltoid, and plantar fascia strain: decreased strain with surgical repair. Plantar force distributions showed medial forefoot offloading with increase laterally.	NR
Sung (2002) [19]	Cadaver	*N* = 13	NR	See Table 4.	Spring ligament length change following MDCO was not different than that for intact foot.	NR

N = number; Y = years; NR = not reported; FDLT = flexor digitorum longus transfer; PTT = posterior tibial tendon; TT = tendon transfer

### Impact on joints pressure

The impact of MDCO on hind- and midfoot joint pressures was analyzed in 10 studies *(*Table 6*)*. Fairbank and colleagues analyzed 11 fresh frozen cadaver legs with an axial load of 700 N which quantified the tibiotalar joint contact characteristics. The flatfoot model was associated with a shift of ankle joint pressure laterally and a quantitative alteration of the contact area compared to the normal ankle. MDCO altered the lateral contact pressure. The authors therefore concluded that MDCO might be a useful alternative to the tibiotalar arthrodesis in cases of early tibiotalar arthritis secondary to varus or valgus hindfoot deformity [25]. Havenhill et al. demonstrated similar results comparing an UCBL orthosis and MDCO in flatfoot cadavers [26]. Davitt and colleagues measured the pressure in the tibiotalar and subtalar joints in a cadaveric study after MDCO. Following a 1 cm MDCO, the average center of force shifted medially in both joints [27]. Steffensmeier and colleagues demonstrated in their cadaveric study that a 1 cm MDCO results in a shift of the pressure distribution 1.58 mm medially in the tibiotalar joint. However, the differences in contact area, mean contact stress, and maximum stress in the tibiotalar joint were not different after MDCO. Similar to the study by Davitt and colleagues [27], in this study, a slight anteromedial shift of the subtalar joint center of pressure was measured [28]. Patrick and colleagues also analyzed subtalar joint force, contact area, and peak contact pressure before and after 1 cm MDCO in 4 cadaveric specimens. After MDCO, there was a slight decrease of subtalar joint force from 211.4 N to 168.8 N, a decrease of contact area from 3.5cm^2^ to 3.1cm^2^, and a decrease of peak contact pressure from 1810 kPa to 1276 kPa. However, all observed changes were not statistically significant [29]. Arangio et al. demonstrated that pathologically increased force in the talo-navicular joint in a flatfoot model almost normalizes after MDCO. They recommended a MDCO in case of a dysfunction of the PTT [30]. Malik and colleagues confirmed these results and recommended a MDCO as an adjunct procedure in talonavicular arthrodesis for patients with a high risk of nonunion [31]. Iaquinto et al. and Spratley et al. proved in a biomechanical model a normalization of joint pressure in the calcaneo-cuboid joint after MDCO [14, 17]. Scott and colleagues and Smith and colleagues examined the effects of MDCO on joint pressures in the hind- and midfoot combined with a lateral column osteotomy [15, 32]. Scott et al. proved with their cadaveric study an increase of lateral forefoot pressures after lateral column lengthening procedures. The addition of MDCO did not lead to an alteration of plantar pressures [32]. Smith and colleagues demonstrated in 6 biomechanical models that the combination of a MDCO with an Evans Osteotomy and a MDCO with a Z-Osteotomy show the greatest amount of correction for both forefoot abduction and hindfoot valgus in a flatfoot model [15].

**Table 6:** Studies reporting of impact of a MDCO on joints pressure

Authors	Type	Feet	Age (Y)	Methods	Results	Consequences
Arangio (2009) [22]	Biomechanical Model	*N* = 1	77	See Table 5.	Flatfoot: Increase in moment talo-navicular joint and joints of 1st and 2nd rays, increasing load on ligaments supporting these joints; moment provided by ligament supporting talo-navicular joint 9.90 Nm; moment provided by ligament supporting naviculo-first cuneiform joint 3.98 Nm; MDCO: moment provided by ligament supporting talo-navicular joint 4.74 Nm; moment provided by ligament supporting naviculo-first cuneiform joint 0.26 Nm.	NR
Davitt (2001) [29]	Cadaver	*N* = 6	NR	Different load in neutral alignment.	1 cm MDCO shifted average center of force 1 mm medially, lateral displacement osteotomy 1.1 mm laterally.	Clinical studies needed to determine if degenerative changes are association with osteotomies.
Fairbank (1995) [27]	Cadaver	*N* = 11	NR	Axial load with 700 N, quantifying tibiotalar joint contact characteristics. Testing in neutral, 10° dorsiflexion and 10° plantarflexion (after 10 mm MDCO or lateral osteotomy). Each of testing series repeated on flatfoot model simulated by soft tissue sectioning.	No difference in tibiotalar joint contact characteristics of all parameters evaluated with foot in plantarflexion. MDCO changes tibiotalar contact area. Flatfoot model shift of pressure laterally and alteration of contact area in all positions compared to normal ankle.	A calcaneal osteotomy may be a useful alternative to tibiotalar arthrodesis in cases of early tibiotalar arthritis secondary to severe varus or valgus deformity.
Havenhill (2005) [28]	Cadaver	*N* = 10	NR	Contact area, contact pressure, peak contact pressure, relative locations of global contact area and peak pressure within ankle joint determined from imprints created on pressure sensitive film. Each limb loaded sequentially under four conditions: intact, flatfoot, flatfoot realigned with UCBL orthosis, and MDCO.	UCBL orthosis and MDCO altered contact characteristics compared with flatfoot condition. Significantly decrease of mean global contact pressure (orthosis > MDCO). Orthosis significantly reduced peak contact pressure. Both interventions significantly corrected lateral shift of center of peak contact pressure.	Clinical management of pes planovalgus with UCBL orthosis or MDCO may avert onset of issues seen with late-stage PTT dysfunction.
Iaquinto (2011) [15]	Biomechanical Model	*N* = 1	NR	Cadaveric anatomy captured by CT imaging and imported to modeling software. Ligament stiffnesses modified to reflect Stage II flatfoot damage, followed by corrective osteotomy. Joint angles, tissue strains, calcaneocuboid contact force, and plantar loads analyzed.	Calcaneocuboid contact load increased 16% from normal to AAFFD conditions. MDCO normalized load.	The degree of deformity in the model suggests that standard sizes for MDCO and lateral column procedures could lead to overcorrection of deformity.
Malik (2020) [32]	Cadaver	*N* = 4	67 (± 8.5)	PTT transection to generate pes planovalgus, arthrodesis of talonavicular joint, 0.5-inch circular force sensing resistor placed within center of talonavicular joint. MDCO 8 mm in neutral position of foot.	Resistance talonavicular joint before MDCO average 388.2 ± 565.9, after MDCO 1016.6 ± 482.7. Force on the talonavicular joint decreased after MDCO.	MDCO beneficial adjunctive procedure in talonavicular arthrodesis for patients with high risk of non-union.
Patrick (2016) [31]	Cadaver	*N* = 8	77.2 (39–89)	Flatfoot model randomly assigned to MDCO (*N* = 4) or calcaneal Z osteotomy (*N* = 4). Load through tibia with 400 N, simultaneous increase in Achilles tendon force to 300 or 500 N. Record of subtalar joint pressures before / after osteotomy.	No statistically significant differences between techniques. After MDCO, decrease of subtalar joint force from 211.4 N (88.3–341.7 N) to 168.8 N (52.0–307.1 N), decrease of contact area from 3.5 cm^2^ (3.3–4.5) to 3.1 cm^2^ (1.4–4.4), and decrease of peak contact pressure from 1810 kPa (848–2475 kPa) to 1276 kPa (1074–2511 kPa).	NR
Scott (2007) [33]	Cadaver	*N* = 16	66 (44–91)	Axial load followed by intact testing, lateral column lengthening, MDCO, Cotton osteotomy. Measured plantar pressures divided into three forefoot regions, two midfoot regions, two hindfoot regions. Analysis of average pressure, peak pressure, contact area.	Lateral column lengthening procedures resulted in increase of lateral forefoot pressures. Addition of MDCO showed no significant alteration of plantar pressures measured after lateral column lengthening alone.	NR
Smith (2017) [16]	Biomechanical Model	*N* = 6	NR	See Table 4.	The combination MDCO and Evans and MDCO and Z procedures significantly increased joint contact force, specifically at calcaneocuboid joint, and ground reaction force along lateral column.	See Table 4.
Steffensmeier (1996) [30]	Cadaver	*N* = 8	NR	Effects of MDCO and lateral displacements of postero-inferior fragment on tibiotalar joint contact mechanics assessed via pressure sensitive film.	1 cm MDCO shifted center of pressure 1.58 mm medially. Global contact parameters not altered by osteotomy. Regional contact parameters changed. Lateral displacements unloaded most medial zone and increased loading of lateral zone.	Translational calcaneal osteotomies may be used to partially offload focal areas of cartilage along the tibiotalar joint.

N = number; Y = years; NR = not reported; FDLT = flexor digitorum longus transfer; PTT = posterior tibial tendon; TT = tendon transfer

### Impact on Achilles tendon

The impact of MDCO on the Achilles tendon was analyzed in 5 of the included studies *(*Table 7*)*. Hadfield and colleagues analyzed the effect of a 1 cm MDCO on Achilles tendon length and plantar foot pressures in 14 cadaveric specimens. The length of the Achilles tendon remained the same after the osteotomy [21]. The same research group analyzed the effect of superior translation of the tuber calcanei with the same parameters. The addition of a 5 mm superior translation did not lead to Achilles tendon lengthening [22]. Nyska and colleagues established an experimental cadaveric AAFFD model by releasing the PTT, spring ligament, and plantar fascia. Applying axial load with a range between 700 and 1400 N substantially aggravated the deformity as confirmed radiographically. Adding a 1 cm MDCO reduced the arch-flattening effect of the Achilles tendon [33]. Arangio et al. proved with their biomechanical model a decreased force provided by the Achilles, flexor hallucis longus (FHL), and flexor digitorum longus (FDL) tendons in AAFFD. Adding a MDCO decreased the force exerted by the Achilles tendon while increasing the force exerted by the peroneus brevis and longus tendons [30]. Sung and colleagues loaded 13 cadaver specimens in a custom testing apparatus. Measurement of PTT force required to achieve early heel rise in an intact foot and were compared to specimens following MDCO and posterior distraction osteotomy. After MDCO, the Achilles force required to achieve the heel rise decreased [18].

**Table 7:** Studies reporting of impact of a MDCO on Achilles tendon

Authors	Type	Feet	Age (Y)	Methods	Results	Consequences
Arangio (2009) [22]	Biomechanical Model	1	77	See Table 5.	AAFFD: Force provided by Achilles tendon, hallucis longus and flexor digitorum longus decreased; MDCO: decreased force exerted by Achilles tendon, increasing force exerted by peroneus brevis and longus.	NR
Hadfield (2003) [23]	Cadaver	14	NR	See Table 5.	No significant increase in Achilles tendon length after MDCO.	NR
Hadfield (2005) [23]	Cadaver	28	64 (48–82)	See Table 5.	Achilles tendon lengthening unchanged in model.	See Table 5.
Nyska (2001) [34]	Cadaver	10	65 (62–72)	Six experimental stages: (1) intact foot without Achilles loading; (2) intact foot with Achilles loading; (3) AAFFD without MDCO and without Achilles loading; (4) AAFFD without MDCO but with Achilles loading; (5) AAFFD with MDCO but without Achilles loading; (6) AAFFD with MDCO and with Achilles loading. AP and lateral radiographs: talonavicular coverage angle, talar-first metatarsal angle, talocalcaneal angle, and height of medial cuneiform.	Between stages 1 and 2, all measurements statistically insignificant. Between stages 3 and 4, for all measurements, Achilles tendon loading aggravated AAFFD. After MDCO (stages 5 and 6), Achilles tendon contributed less to arch flattening. In AAFFD, loading of Achilles tendon increases deformity. MDCO significantly decreases arch flattening effect of tendon and limits potential increase of the deformity.	MDCO alternative to tibiotalar arthrodesis in cases of early tibiotalar arthritis secondary to hindfoot deformity.
Sung (2002) [19]	Cadaver	13	NR	See Table 4.	Osteotomies reduced Achilles force required to achieve heel rise position.	NR

N = number; Y = years; NR = not reported; AP = antero-posterior

### Impact on gait pattern

The impact on gait pattern was analyzed in 3 studies *(*Table 8*)*. Marks and colleagues analyzed gait parameters as well as radiographic alignment in 14 patients with MDCO and six patients with lateral lengthening osteotomy of the calcaneus. In both groups, a significant improvement of all gait parameters was observed. The MDCO group demonstrated improved first ray plantarflexion, while the lateral lengthening group presented with better heel inversion [34]. Michelson and colleagues examined the alteration in ankle motion after MDCO. At maximal dorsiflexion, internal rotation and varus motion increased. There was no significant difference in plantar flexion. Therefore, they hypothesized that a MDCO may predispose to premature ankle arthritis as a consequence of the altered ankle mechanics [35]. In a clinical study by Schuh et al., the authors examined 75 feet of 73 patients with posterior tibial tendon dysfunction (PTTD) stage II who underwent an FDL tendon transfer and MDCO. Plantar pressure distribution and American Orthopaedic Foot and Ankle Society (AOFAS) score were analyzed 48 months after surgery. The authors saw statistically significant correlations between the AOFAS score and loading parameters of the medial midfoot [36].

**Table 8:** Studies reporting of impact of a MDCO on gait parameter

Author	Type	Feet	Age (Y)	Methods	Results	Consequences
Marks (2009) [35]	Clinical	*N* = 21	52.4 (± 9.1)	Patients with AAFFD evaluated before and after reconstruction (flexor digitorum longus substitution combined with MDCO or flexor digitorum substitution with lateral column fusion or osteotomy). Foot/ankle kinematics and temporal spatial parameters analyzed using Milwaukee Foot Model.	Significant improvement of all gait parameters. MDCO: improved 1st ray plantarflexion; lateral lengthening group: better heel inversion. Both procedures demonstrated comparable improvements in radiographic measurements.	Lateral column procedures tend to create greater radiographic improvement, but with a higher incidence of soft tissue and hardware complications.
Michelson (1998) [36]	Cadaver	*N* = 8	NR	Examination of alteration in ankle motion after MDCO. Prevention of motion of all foot joints but ankle and subtalar joint motion.	At maximal dorsiflexion 76% increase in internal rotation and increase of 425% in hindfoot varus for intact ankles. No significant differences in plantar flexion.	MDCO may predispose to premature ankle arthritis as consequence of altered ankle motion.
Schuh (2013) [37]	Clinical	*N* = 75	59.9 (43–79)	Patients with PTTD stage II, flexor digitorum TT, and MDCO. Plantar pressure distribution and AOFAS score 48 months after surgery. Pedobarographic parameters: lateral and medial force index of the gait line, peak pressure (PP), maximum force (MF), contact area (CA), contact time (CT) and force time integral (FTI).	In lesser toe region PP, MF, CT, FTI and CA reduced, in forefoot region MF increased. Statistically significant correlations between AOFAS score and loading parameters of medial midfoot. Flexor digitorum TT and MDCO: Impaired function of lesser toes during stance phase with compensating increased load in forefoot region.	Reconstructive procedures for PTTD should aim to decrease loading parameters at the midfoot region.

N = number; Y = years; NR = not reported; TT = tendon transfer; AOFAS = American Orthopaedic Foot and Ankle Society; PTTD = posterior tibial tendon dysfunction

The main biomechanical consequences of the MDCO are summarized in Table 9.

**Table 9:** Main biomechanical consequences of the MDCO

	Biomechanical consequences
Plantar fascia	- drop of plantar fascia strain - decrease of plantar fascia length - no impact of plantar fascia release on corrective potency following MDCO
Medial longitudinal arch	- no change of spring ligament length - no impact of spring ligament reconstruction on outcome of MDCO - shift of excess forces laterally - decreased load on medial longitudinal arch
Joints pressure	- shift of the average center force medially in tibiotalar and subtalar joints - decrease of subtalar and tibiotalar joint force, decrease of the contact area, decrease of peak contact pressure
Achilles tendon	- no impact of MDCO on Achilles tendon length - reduction of arch-flattening effect of Achilles tendon
Gait pattern	- improvement after MDCO - impaired function of the lesser toes during stance phase of walking with increased load of forefoot - MDCO may predispose to premature ankle arthritis as a consequence of altered ankle motions

## Discussion

27 studies assessing the biomechanical impact of MDCO on foot function were included in the present systematic review. We found evidence that MDCO impacts function of the plantar fascia, integrity of the medial longitudinal arch, alters peritalar joint pressures, and has an influence on Achilles tendon function. Consequently, specific gait pattern parameters were found to be impacted by MDCO.

Interestingly, our analysis showed an increase of plantar fascia strain in the case of AAFFD with a drop following MDCO [14–17]. In addition, Sung et al. reported a decrease of plantar fascia length after MDCO [18]. Another study showed no impact of a plantar fascia release on the corrective potential following MDCO [13]. Therefore, an additional release of the plantar fascia may not be effective in cases where MDCO is performed.

MDCO does not change spring ligament length, while spring ligament reconstruction may not impact the expected outcome following MDCO [18, 19, 23]. However, a substantial shift of excess forces towards the lateral aspect of the longitudinal arch was found in several studies [17, 20–22, 30], reflecting a high strength of evidence. Resnick et al. additionally analyzed deltoid ligament force under axial load and concluded that MDCO in combination with a triple arthrodesis may be a viable treatment option when the hindfoot cannot be positioned properly following fusion procedures [24]. MDCO may not only correct hindfoot valgus deformity, but also offload the medial aspect of the foot and therefore can serve as a solution after hindfoot fusion procedures with non-union.

The studies showed a shift of the average center of force medially in the tibiotalar and subtalar joints following MDCO [27, 28]. Additionally, an overall decrease of subtalar and tibiotalar joint forces, contact area, and peak contact pressures were evident in several studies [29, 30]. These aspects of impact on joint pressure show a high strength of evidence. MDCO can therefore be recommended in case of a tibialis posterior tendon dysfunction or as an additional procedure in talonavicular arthrodesis for patients with a high risk of non-union [30, 31]. In addition, Iaquinto et al. and Spratley et al. showed a normalization of increased joint pressures in the calcaneo-cuboid joint after MDCO [14, 17].

Of note, studies have shown MDCO to not impact Achilles tendon length [21, 22], while a reduction of the arch-flattening effect of the Achilles tendon was evident in two other studies [30, 33]. Additionally, a decrease in force at the level of the Achilles tendon was required to achieve heel rise position after MDCO [18]. Consequently, an additional lengthening of the Achilles tendon when performing MDCO for PCFD reconstruction may not be required in most patients.

Specific gait pattern parameters showed significant improvement after MDCO [34]. However, in a clinical study, Schuh et al. showed that MDCO impairs function of the lesser toes during the stance phase of walking, with an increased load of the forefoot [36]. In addition, Michelson and colleagues hypothesized that MDCO may predispose to premature ankle arthritis as a consequence of altered ankle mechanics [35]. The studies evaluating gait parameters showed a moderate strength of evidence, showing the need for further research in this field. The negative effects on gait pattern parameters must be considered when MDCO is performed. Afterall, the indication for MDCO must be thoroughly evaluated to avoid these postoperative complications. Weightbearing computed tomography as a newer preoperative diagnostic tool, which presents the possibility of imaging in the physiological standing position to assess for forefoot and hindfoot alignment, could reduce the risk of negative effects of MDCO in the future [37–43].

Within the included studies, 18 (66,67%) make statements about the predictive value or operative consequences of the biomechanical findings, showing that knowledge about the biomechanical consequences of MDCO is important in understanding the effects of the osteotomy on the entire foot.

The presented study has some limitations. First, our findings are limited by the quality of the included studies. However, this is an inevitable limitation of systematic literature reviews and meta-analyses in general. For example, most of the included studies failed to report the educational level of the researchers. Also, most studies did not mention whether the observations were performed by one or more researchers, a possible source of bias. Second, only studies written in English were considered, excluding valuable contributions written in other languages.

## Conclusion

A thorough knowledge of how MDCO impacts foot function is key in properly understanding the postoperative effects of this commonly performed procedure. There is evidence that MDCO effects the plantar fascia, medial longitudinal arch, peritalar joint pressures, Achilles tendon, and consequently specific gait pattern parameters. Future research should consider newer diagnostic tools including weightbearing computed tomography.

### Electronic supplementary material

Below is the link to the electronic supplementary material.

Supplementary Material 1
