# Supplementary material for: Impact of the medial displacement calcaneal osteotomy on foot biomechanics: a systematic literature review
Source: Arch Orthop Trauma Surg. 2024 Mar 30;144(5):1955–67. doi: 10.1007/s00402-024-05267-9 (PMC11093820; doi:10.1007/s00402-024-05267-9)
Supplement: Supplementary file 1 — Supplementary Material 1 [file 402_2024_5267_MOESM1_ESM.pdf]

(Form2)

Conflict of Interest Self-Declaration Form for All Authors

Names of All Authors: Gunnar Mueller, Karl-Heinz Frosch, Alexej Barg, Carsten Schlickewei, Hanneke Weel, Nicola Krähenbühl, Matthias Priemel, Elena Mueller

Title of Manuscript: Impact of the Medial Displacement Calcaneal Osteotomy on Foot Biomechanics: A Systematic Literature Review

（Starting from a year before the manuscript submission, all corresponding authors must self-declare COI status of each author with companies or organizations related to the contents of presentation for the past three years.）

Criteria	Applicability	If applicable, provide company name, etc.
1. Appointment to official, advisory or employee position of companies or for-profit organizations<br>Annual compensation of one million yen or more from one company or organization	Yes ・ No	
2. Ownership of stock or equity including new share subscription rights<br>Annual profit of more than 1 million yen from one company, or holding stock of 5% or more	Yes ・ No	
3. Patent royalties or licensing fees from company or for-profit organization<br>One annual royalty fee of 1 million yen or more	Yes ・ No	
4. Honoraria (such as lecture fees) from company or for-profit organization paid as compensation for the time and/or labor of a researcher engaged in conference attendance (e.g., presentation, advice, etc.)<br>Annual fee of 500,000 yen or more from one company or organization	Yes ・ No	
5. Manuscript fees paid by a company or for-profit organization as compensation for writing a pamphlet or other publication<br>Annual fee of 500,000 yen or more from one company or organization	Yes ・ No	
6. Research funding provided by company or for-profit organization<br>Annual total amount of 1 million yen or more paid for medical research (collaborative research, contract research, etc.) from one company or organization	Yes ・ No	
7. Scholarship to the declarer provided by company or for-profit organization<br>Annual total amount of 1 million yen or more paid to the declarer or the affiliated institution from one company or organization	Yes ・ No	
8. Endowed chair provided by company or others<br>In case of affiliation with companies, etc.	Yes ・ No	
9. Other remuneration (e.g., travels not directly related to research, gifts, etc.)<br>Annual compensation of 50,000 yen or more from one company or organization	Yes ・ No	

（This form will be stored for 3 years from the time of publication.）

Date of Disclosure: 06.04.23

Signature of Corresponding Author: E. Müller

(Supplementary Form 2)

Conflict of Interest Self-Declaration Form for Individual Authors

Name of Author: ______________________________

Title of Manuscript: ______________________________

(Starting from a year before the manuscript submission, all authors must self-declare COI status with regard to any companies or organizations related to the contents of presentation for the past three years.)

Criteria	Applicability	If applicable, provide company name, etc.
1. Appointment to official, advisory or employee position of companies or for-profit organizations<br>Annual compensation of one million yen or more from one company or organization	Yes ・ No	
2. Ownership of stock or equity including new share subscription rights<br>Annual profit of more than 1 million yen from one company, or holding stock of 5% or more	Yes ・ No	
3. Patent royalties or licensing fees from company or for-profit organization<br>One annual royalty fee of 1 million yen or more	Yes ・ No	
4. Honoraria (such as lecture fees) from company or for-profit organization paid as compensation for the time and/or labor of a researcher engaged in conference attendance (e.g., presentation, advice, etc.)<br>Annual fee of 500,000 yen or more from one company or organization	Yes ・ No	
5. Manuscript fees paid by a company or for-profit organization as compensation for writing a pamphlet or other publication<br>Annual fee of 500,000 yen or more from one company or organization	Yes ・ No	
6. Research funding provided by company or for-profit organization<br>Annual total amount of 1 million yen or more paid for medical research (collaborative research, contract research, etc.) from one company or organization	Yes ・ No	
7. Scholarship to the declarer provided by company or for-profit organization<br>Annual total amount of 1 million yen or more paid to the declarer or the affiliated institution from one company or organization	Yes ・ No	
8. Endowed chair provided by company or others<br>In case of affiliation with companies, etc.	Yes ・ No	
9. Other remuneration (e.g., travels not directly related to research, gifts, etc.)<br>Annual compensation of 50,000 yen or more from one company or organization	Yes ・ No	

（This form will be stored for 3 years from the time of publication.）

Date of Disclosure:

Signature of Author: ______________________________
